# Use of Real-World Data and Machine Learning to Screen for Maternal and Paternal Characteristics Associated with Cardiac Malformations

**DOI:** 10.21203/rs.3.rs-4490534/v1

**Published:** 2024-06-11

**Authors:** Jeremy Brown, Krista Huybrechts, Loreen Straub, Dominik Heider, Brian Bateman, Sonia Hernandez-Diaz

**Affiliations:** Harvard T.H. Chan School of Public health; Brigham and Women’s Hospital; Brigham and Women’s Hospital; Heinrich-Heine-University of Düsseldorf; Brigham and Women’s Hospital; Harvard University

## Abstract

Effective prevention of cardiac malformations, a leading cause of infant morbidity, is constrained by limited understanding of etiology. The study objective was to screen for associations between maternal and paternal characteristics and cardiac malformations. We selected 720,381 pregnancies linked to live-born infants (n=9,076 cardiac malformations) in 2011–2021 MarketScan US insurance claims data. Odds ratios were estimated with clinical diagnostic and medication codes using logistic regression. Screening of 2,000 associations selected 81 associated codes at the 5% false discovery rate. Grouping of selected codes, using latent semantic analysis and the Apriori-SD algorithm, identified elevated risk with known risk factors, including maternal diabetes and chronic hypertension. Less recognized potential signals included maternal fingolimod or azathioprine use. Signals identified might be explained by confounding, measurement error, and selection bias and warrant further investigation. The screening methods employed identified known risk factors, suggesting potential utility for identifying novel risk factors for other pregnancy outcomes.

## Introduction

Cardiac malformations are one of the most common congenital malformations and a major cause of infant mortality and lifelong morbidity.^1,2,3^ Effective prevention, which could reduce this burden, is hampered by limited understanding of the etiology of these malformations outside of known genetic causes.^3^

Several non-genetic potential causes of cardiac malformations have been identified including maternal clinical conditions and medications, such as diabetes and oral retinoids.^4^ Previous studies have primarily, though not exclusively,^5^ focused on single exposures or have been limited by the data collection instruments to a restricted set of pre-defined risk factors.^6,7^ Occasionally, clinical observation of unusual clusters might provide clues (e.g., Ebstein’s anomaly after lithium exposure), however, it can also trigger false alarms.^8–10^ Furthermore, while there has been some suggestion that paternal exposures preceding conception may also lead to congenital malformations, there is little evidence to date, including for cardiac malformations.^11,12^ Despite cardiac malformations being the most common malformations, given their estimated prevalence of ~ 1% among live births and their multifactorial etiology, it may be helpful to complement passive clinical surveillance and *ad hoc* studies of specific exposures by screening of large healthcare utilization databases.

The aim of this study was to identify and characterize associations of both maternal and paternal clinical conditions and medications with cardiac malformations among infants using statistical and machine learning methods in a large cohort of pregnancies linked to live-born infants in insurance claims data, Merative MarketScan Commercial Claims and Encounters (CCAE), from the United States (US).

## Results

We selected a cohort of 720,381 pregnancies linked to live births occurring among 647,711 mothers for the analysis of maternal associations. Multiple gestation occurred in 24,631 (3.4%) of pregnancies. For the analysis of paternal associations, we linked 507,442 (70.4%) of the 720,381 pregnancies to one of 457,096 fathers. The median age at pregnancy for mothers was 31 years (interquartile range [IQR] 28–34) and for fathers 33 years (IQR 30–37).

Among the 720,381 pregnancies, there were 9,076 pregnancies in which one or more infants had a cardiac malformation (1.3%). The most common cardiac malformations (see Supplementary Table 1) were ventricular septal defects (n = 5,349, 0.7%), left-sided defects (n = 1,316, 0.2%), and atrial septal defects (n = 940, 0.1%).

### Screening for associations

**Figure 1:**
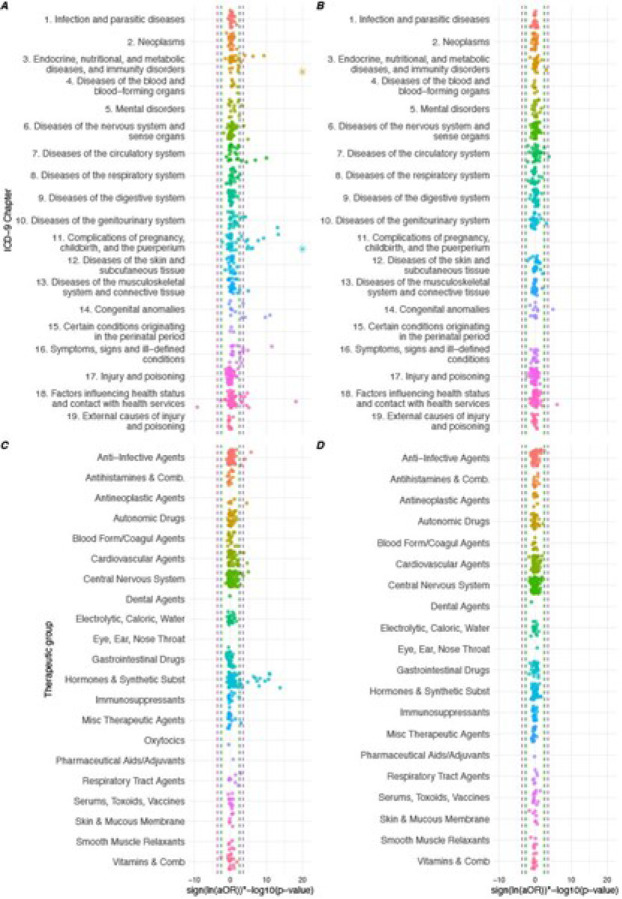
Manhattan plot of associations between congenital heart defects and A) maternal clinical diagnoses, B) paternal clinical diagnoses, C) maternal medications, and D) paternal medications Definitions: FDR, false discovery rate; RD, risk difference; Misc, miscellaneous. Note: asterisks represent -log10(p-values) greater than 20.

Screening of associations with derived covariates selected, at the 5% FDR threshold, 49 associations with maternal diagnoses, 5 associations with paternal diagnoses, 27 associations with maternal medications, and no association with paternal medications (Fig. 1 and Box 1). Of the 81 selected associations, 1 variable had reduced odds and 80 increased odds of cardiac malformations. The adjusted odds ratio was > 2 for 22 associations. At a 1% FDR 56 associations were selected and at 10% FDR more associations (n = 101) were selected.

### Characterization of associations

**Figure 2:**
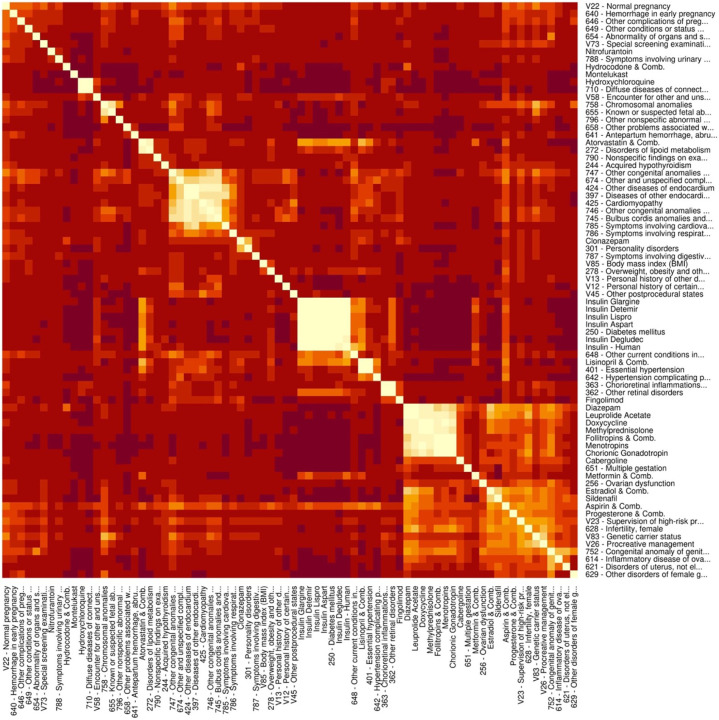
Similarity of latent vector representation of clinical and medication codes identified in the mother (lighter color equals more similar)

**Figure 3:**
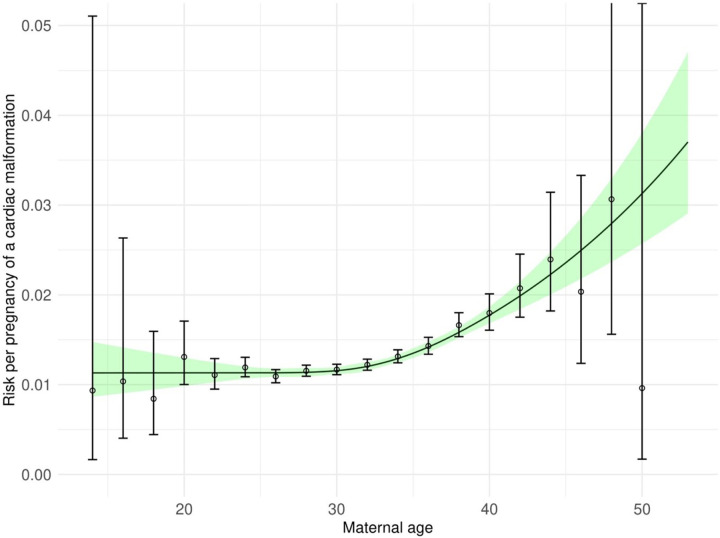
Maternal age and per pregnancy predicted risk of a cardiac malformation Note: circles represent point estimates for risk for two-year intervals where the number of pregnancies in that age group exceeded 100.

Manual categorization of selected codes highlighted groups of codes relating to maternal diabetes, maternal obesity, other maternal cardiometabolic conditions, cardiac conditions, fertility treatment and multiple gestation, prenatal diagnoses of malformations and malformations in the parent, and obstetric conditions (Box 1). Automated identification of groups using hierarchical clustering after latent semantic analysis similarly identified groups relating to diabetes, dyslipidemia, chronic hypertension, fertility treatment and prenatally diagnosed/parental malformations (Fig. 2 and Table 1). Latent semantic analysis highlighted links between codes that are not immediately apparent from inspection, such as the relation between doxycycline, methylprednisolone, diazepam, and leuprolide acetate, which are all used in assisted reproductive technology (ART). Elevated maternal age was associated with increased risk of cardiac malformations (Fig. 3).

### Identification of high-risk subgroups

Application of the Apriori-SD algorithm selected subgroups at elevated risk pertaining to the maternal characteristics of pregestational diabetes, chronic hypertension, multiple gestation, and prenatally diagnosed known or suspected malformations (Table 2). Identified subgroups at elevated risk were defined by the conjunction of two or fewer codes, which may relate to limited power to identify smaller subgroups defined by 3 + codes.

### Secondary analyses

Secondary analyses with specific cardiac malformations (Supplementary Tables 2–9) selected at the 5% FDR were associations between diabetes and antidiabetic medications and increased atrial septal, conotruncal, left-sided, and ventricular septal defects. Other associations selected included an association between clonazepam and increased left-sided defects, an association between screening for viral or chlamydial infections and increased defects of the great cardiac vein, and vitamin B deficiency and increased other cardiac malformations. No associations were identified with single ventricular, atrioventricular septal, patent ductus arteriosus (PDA), or persistent pulmonary hypertension of the new-born (PPHN). However, numbers of these outcomes were small limiting power to detect associations.

### Sensitivity analyses

Exclusion of pregnancies with chromosomal abnormalities led to the identification of similar associations to the main analysis, though a few additional associations were selected leading to 90 selected associations at a 5% FDR. Associations with hypertension and hyperlipidemia attenuated after restricting to women without diabetes (Supplementary Table 10), though an association with essential hypertension remained (aOR 1.26, 95% CI 1.11–1.44). Associations with fertility treatments attenuated after restricting to singletons (Supplementary Table 10). Associations with many other medications attenuated after restricting to singleton pregnancies amongst women without diabetes highlighting the use of these medications in fertility treatment and diabetes (e.g., methylprednisolone, cabergoline, diazepam, and doxycycline in assisted reproductive technology; aspirin prophylaxis to prevent preeclampsia in pregnancies with multiple gestation or diabetes; diabetes concomitant with hypertension treated with lisinopril). After further restricting to pregnancies without a malformation recorded in the parent prior to last menstrual period (LMP), associations with parental cardiac condition attenuated (Supplementary Table 10). After restriction, strong associations with medications (aOR > 2) remained for azathioprine and fingolimod. Requiring two or more codes to define presence led to the additional selection of the following associations (Supplementary Table 10): other known or suspected fetal and placental problems affecting management of mother, number of fetuses, tramadol, disorders of sweat glands, varenicline, and vitamin D deficiency. Varying the time window of assessment for clinical covariates to acute exposures (first trimester among mothers, 90 days prior to LMP for fathers) led to the selection of many of the same associations, with the supplementary addition of disorders of mineral metabolism and paternal gingival and periodontal diseases. Fewer, but similar, associations to the main analysis were selected when using 3- and 4-digit ICD-9 and ICD-10 codes in analyses stratified by ICD code period (Supplementary Tables 11–14).

## Discussion

In this study we applied statistical and machine learning methods to screen for associations between maternal and paternal characteristics and cardiac malformations. Screening identified both known risk factors including pregestational diabetes, chronic hypertension, obesity, and parental malformations in addition to less well-recognized potential signals including maternal use of fingolimod and azathioprine. Increased risk of cardiac malformations in the offspring of women with pregestational diabetes has long been recognized and has been found to increase with HbA1c levels.^6,13–15^ Patients with advanced or uncontrolled diabetes, such as women with type 2 diabetes requiring insulin or other second line therapies, have the highest risks.^16^ Suggested mechanisms include hyperglycemia-induced oxidative stress, altered signaling pathways, and epigenetic modifications.^17^ Although finding an association is not surprising, the prominent role of diabetes-related factors on the risk of cardiac malformations highlights the need for public health action to prevent incidence and progression of diabetes before pregnancy.

Chronic hypertension was associated with an increased risk of cardiac malformations even after restricting to pregnancies among mothers without recorded pregestational diabetes. A previous study using nationwide Medicaid data, found an association between both treated and untreated chronic hypertension and cardiac malformations after adjustment for potential confounders (treated hypertension OR 1.6, 95% CI 1.4–1.9 and untreated hypertension OR 1.5, 95% CI 1.3–1.7).^18^ The mechanism of increased risk is not at present fully comprehended.^18,19^

Maternal obesity was found to be associated with cardiac malformations including after restricting pregnancies to mothers without recorded pregestational diabetes. Higher maternal BMIs have been consistently associated with increased malformations, including cardiac malformations.^20,21^ Potential mechanisms include elevated glycemic levels without diagnosed diabetes^22^, nutritional deficiencies^23^, and reduced prenatal detection among obese women and hence reduced termination of pregnancy due to fetal anomaly.^24,25^

The finding of increased risk with both paternal and maternal recording of cardiac malformations may relate to familial inheritance through genetic or shared environmental risk factors.^26^ Another explanation for the association may be the prenatal diagnosis of syndromic defects such as Down’s syndrome early in pregnancy coded in maternal claims records.^27^ Although prenatal screening of anomalies is typically conducted at 15 weeks and we considered diagnosis in the first 12 weeks, we might still be including early diagnoses if our LMP was slightly miscalculated in some instances. The association with paternal malformations suggests that the association is not exclusively due to prenatal diagnosis of the outcome.

While fertility treatments were associated with increased risk of cardiac malformations in our study, this may be an artefact of counting outcomes by presence per pregnancy. In MarketScan CCAE data it is difficult to distinguish individual infant outcomes within a multiple gestation pregnancy and as such we counted presence of malformations at the pregnancy-level. Counting at the pregnancy-level can lead to up to 2-times higher risk in twin pregnancies even if the individual fetal risk is not elevated in twins.^28^ After restricting to singleton gestation pregnancies, associations with fertility treatments were attenuated and close to the null.

The identification of known risk factors using these statistical and machine learning methods indicates the potential value of these methods for identifying novel associations. A challenge to the application of machine learning methods in insurance claims and electronic health record data is the sparsity of the data (i.e., a large fraction of values are zero), the high degree of correlation between variables, and the interpretability of results.^29,30^ Interpretable unsupervised machine learning methods, for example the dimensionality reduction technique of latent semantic analysis and the rule-based machine learning algorithm Apriori-SD, can handle sparse correlated data and aid in the interpretation of findings, such as in this study by highlighting the use in common of doxycycline, methylprednisolone, and diazepam in ART. There can be many explanations for identified associations, including latent characteristics of those with the code (e.g., diabetes among those with a code for insulin), therefore characterizing the relation between codes can aid in interpretation.

Less recognized signals identified included maternal use of fingolimod and azathioprine. These associations may be due to the characteristics of women prescribed these medications, but nevertheless highlight the need for further evaluation of the safety in pregnancy of the different medications for the indications for which these medications are prescribed (i.e., multiple sclerosis, autoimmune disease). Both fingolimod and azathioprine have been found in animal studies involving rats and rabbits to be teratogenic, including at doses equivalent to the human dose.^31,32^

An association was selected between clonazepam and left-sided defects. The association between benzodiazepines and congenital malformations has been widely investigated, but findings have been conflicting, and the association may have been subject to residual confounding and recall bias.^33–35^ In our study, the association between diazepam, which is used as a uterine relaxant in ART, attenuated after restricting to singletons. Nonetheless, the association between specific benzodiazepines and cardiac malformations, specifically left-sided defects, deserves further evaluation.

There was a relative absence of associations with paternal exposures. Few associations with paternal non-genetic exposures have been previously identified. A recent exception is a cohort study that reported an association between paternal metformin use and genital defects.^11^

The strengths of this study include the large cohort size, validated outcome (with PPV of 78% in a validation study^36^), examination of risk with subtypes of cardiac malformation, linkage to fathers, and careful control for multiple testing.

While the cohort is relatively large, associations with rare exposures may not be detected, though these will be arguably of less clinical significance given their infrequency. The study is limited to exposures that can be captured in insurance claims data, which includes both prescription medications and clinical diagnoses, but excludes genetic variables, lab measurements, dietary exposures, and other lifestyle factors. Known or suspected teratogens such as oral retinoids are unlikely to be dispensed in pregnancy and, therefore, unlikely to be detected. Furthermore, pregnancies exposed to known teratogens are more often terminated. The study was restricted to live births, introducing the possibility of selection bias, for example due to differential termination of pregnancy for fetal anomaly.^37^ Given that our aim was not to determine causality, but rather to identify associations for further investigation, the identified associations should not be interpreted as causal. Both the detection and the absence of specific signals can be explained by random and systematic errors such as confounding, information and selection biases.

In conclusion, through screening of associations aided by unsupervised machine learning methods, we identified a number of characteristics associated with cardiac malformations. Some associations were known, and some represent potential signals. The ability of the screening methods employed to detect both known and suspected risk factors for cardiac malformations, suggest potential utility of these methods in identifying novel risk factors for other malformations and other adverse birth outcomes.

## Methods

### Data sources

Data were obtained for years 2011–2021 of Merative MarketScan CCAE, a US commercial insurance claims dataset. MarketScan CCAE is one of the largest US nationwide datasets of commercial health beneficiaries and contains deidentified inpatient and outpatient healthcare utilization data including diagnoses, procedures, and all outpatient pharmacy dispensed prescription medications.^38^

### Study population

We have previously defined a cohort of 2.7 million pregnancies linked to infants in MarketScan CCAE. Algorithms developed to identify pregnancies, link to infants, assign a pregnancy outcome, and estimate the date of LMP have previously been described in detail.^39–42^

For the present study we included all pregnancies among mothers aged 12–55 years linked to live-born infants meeting the following eligibility requirements: First, in order to ascertain exposures and pregnancy outcomes, all mothers were required to be continuously enrolled from at least 180 days before LMP to end of pregnancy plus 30 days. Second, mothers were required to have full medication benefits from LMP minus 180 days to end of pregnancy in order to characterize medication exposures. Third, to ascertain cardiac malformations, linked infants were required to have enrolment to at least 90 days following the end of pregnancy except in cases of infant death.

To identify associations with paternal exposures we identified a cohort of fathers linked to both pregnant women and to live-born infants through family case number and year of delivery. Fathers were required to have continuous enrolment and medication benefits from the mother’s LMP minus 180 days to LMP in order to ascertain exposures during spermatogenesis.

### Exposures

Indicator variables for maternal and paternal clinical conditions and medications were derived based on an adaptation of methodology employed in the high-dimensional propensity score algorithm.^43^

For maternal medication exposure, we created indicator variables for dispensation of one or more prescriptions, categorized by RED BOOK ^™^ therapeutic detail code, within the first trimester (LMP to LMP plus 90 days), a critical period during embryogenesis for the development of structural abnormalities of the heart.^44^ Therapeutic detail codes categorize medications at the level of the generic ingredient. For paternal exposure, indicator variables were created for medication dispensation, categorized by therapeutic detail code, during spermatogenesis (LMP minus 90 days to LMP).

For maternal clinical diagnoses, indicator variables were created based on International Statistical Classification of Diseases and Related Health Problems (ICD), 9th revision (ICD-9; before October 2015) and 10th revision (ICD-10; October 2014 onwards), codes. ICD-9 codes were categorized by three digit ICD-9 section code, a categorization that has proven useful in the high-dimensional propensity score.^43^ ICD-10 codes were mapped to the respective ICD-9 3-digit section code using General Equivalence Mappings provided by the Centers for Medicare & Medicaid Services (see Supplement for details).^45^ Code occurrence was assessed between LMP minus 180 days and LMP plus 90 days in mothers and between LMP minus 180 days and LMP among fathers.

Separately among mothers and fathers, we selected the 500 most common medications dispensed and 500 most common ICD-9 section codes occurring within the exposure assessment windows.

### Outcomes

The primary outcome was cardiac malformation in the live-born infant defined by ICD-9 and ICD-10 codes using code lists and algorithms previously validated by chart review (see Supplement for details of algorithm).^36,46^ Secondary outcomes were the following specific cardiac defects: conotruncal, single ventricle, ventricular septal, atrial septal, atrioventricular septal, right-sided, left-sided, patent ductus arteriosus (PDA), persistent pulmonary hypertension of the new-born (PPHN), great cardiac veins, and other cardiac defect (see Supplement for code lists).^2^

### Statistical and machine learning analyses

#### Screening for associations

To identify associations, in the separate maternal and paternal data logistic regression models were separately fitted for each code adjusting for maternal age using restricted cubic splines. We adjusted for maternal age, in order to identify associations independent of this known risk factor.^47^ P-values were calculated using likelihood ratio tests.^20^Associations were identified at 1%, 5%, and 10% false discovery rate (FDR) using the Benjamini-Hochberg procedure to account for multiple testing.^48^ We applied a bootstrap-based bias-correction method to correct adjusted odds ratios for the winner’s curse effect, in which effect estimates selected by screening are artificially elevated due to the selecting associations with the smallest p-value.^49^ Log-10 p-values were plotted using a variant of the Manhattan plot, in which we multiply the p-value by the sign of the log odds ratio to distinguish positive from negative associations.

#### Characterization of associations

To characterize identified associations, in order to help generate hypotheses of underlying causes, we used latent semantic analysis.^50^ Latent semantic analysis is an unsupervised machine learning method from natural language processing, in which a lower dimensional representation of the data is derived assuming the observed high-dimensional data is a function of a smaller number of underlying latent factors.^51–53^ In this lower dimensional representation, the vector representation of terms that occur in similar contexts will be close in distance. For example, diabetes medications, diabetes diagnoses, and sequelae of diabetes, such as diabetic retinopathy, are all indicators of a latent state of diabetes.

To perform latent semantic analysis, truncated singular value decomposition (SVD) was performed on the *n*×*d* matrix for the *n* individuals and *d* dimensions for all mapped ICD-9 section codes and therapeutic detail codes.^52^ Per individual counts for each code were log transformed before SVD. After SVD, the 500 dimensions with the largest singular values were retained. To characterize the relationship between variables identified through screening, the similarity of the vector representation of these identified codes in lower dimensional space was calculated using cosine similarity.^52^ For visualization and grouping, agglomerative hierarchical clustering was used to cluster codes into groups of similar variables based on angular distance between vector representations.^54^ In addition to this data-driven grouping of codes, we conducted a separate manual categorization of codes into groups with clinical similarity (e.g., diabetes medications and diagnoses).

To characterize the association between maternal age and cardiac malformations we plotted the predicted values from a logistic regression model fitted using a restricted cubic spline for maternal age.

#### Identification of high-risk subgroups

To identify high-risk subgroups of pregnancies characterized by the conjunction of multiple conditions, the Apriori-SD (subgroup discovery) rule-based machine learning algorithm was applied.^55^ This algorithm has two components. First, the Apriori algorithm exhaustively generates all rules of the form {X, Z} = > Y (e.g., {chest pain, faint, shortness of breath} = > myocardial infarction) of length (i.e., number of variables in the left-hand side of the rule) less than a specified maximum, with a minimum support (i.e., proportion of the population with X, Y, and Z), and with a minimum confidence (i.e., proportion of those with X and Z who have Y).^54,56^ Second, those rules with the desired target in the right hand side (i.e., cardiac malformation) are processed to identify distinctive subgroups in the population at high risk of the outcome.

The Apriori-SD algorithm was applied to the binary maternal data as defined in the Exposures section for all mapped ICD-9 section codes and therapeutic detail codes. Random down-sampling of non-cases was applied to ensure class balance (i.e., 50% with the outcome and 50% without) and improve algorithm performance.^52^ To generate rules with support in the data, the minimum support was set at 0.1%, minimum confidence at 55%, and the maximum length of the left-hand side of the rule at 10 variables. Age was categorized for this analysis into < 35 years and ≥ 35 years. The Westfall-Young permutation testing procedure was applied to select rules while controlling the family-wise error rate at 5%.^29^ Prevalence of subgroups and risk ratios between subgroup membership and the outcome were calculated in the entire dataset without undersampling.

### Secondary and sensitivity analyses

As a secondary analysis, screening of associations was conducted separately for the secondary outcomes.

Sensitivity analyses for screening of associations were 1) restriction to pregnancies without chromosomal abnormalities given these have predefined definite causes 2) restriction to singletons since pregnancies with more than one fetus would by definition have a higher probability of having at least one fetus with a diagnosis; 3) restriction to singleton pregnancies among mothers without diabetes to ascertain associations independent of diabetes (a known risk factor for cardiac malformations) or multiple gestation, 4) restriction to singleton pregnancies to mothers without diabetes and parents without a congenital malformation recorded in the 6 months prior to LMP to ascertain associations independent of maternal diabetes, multiple gestation or parental malformation, 5) conducting analyses separately in data pre-October 2015 using ICD-9 section codes and from October 2015 onwards using 3-digit ICD-10 categories to assess the sensitivity of results to mapping of ICD-10 codes to ICD-9 sections, 6) conducting analyses separately in data pre-October 2015 using 4-digit ICD-9 codes and from October 2015 onwards using 4-digit ICD-10 codes, 7) definition of indicator variables by two or more occurrences within the assessment window to increase specificity of exposure classification, and 8) varying the assessment window for clinical covariates to capture with more specificity acute clinical exposures (e.g., infections) to LMP to LMP plus 90 days among mothers and to LMP minus 90 days to LMP among fathers.

## Figures and Tables

**Table 1:** Code groupings identified in latent semantic analysis

Code terms	Number of code terms in group
Diabetes mellitus; Insulin - Human; Insulin Aspart; Insulin Degludec; Insulin Detemir; Insulin Glargine; Insulin Lispro	7
Disorders of lipoid metabolism; Atorvastatin & Comb.	2
Other retinal disorders; Chorioretinal inflammations, scars, and other disorders of choroid	2
Diseases of other endocardial structures; Other diseases of endocardium; Cardiomyopathy; Bulbus cordis anomalies and anomalies of cardiac septal closure; Other congenital anomalies of heart	5
Essential hypertension; Lisinopril & Comb.	2
Known or suspected fetal abnormality affecting management of mother; Chromosomal anomalies	2
Diffuse diseases of connective tissue; Hydroxychloroquine	2
Leuprolide Acetate; Diazepam; Methylprednisolone; Doxycycline	4
Chorionic Gonadotropin; Menotropins; Follitropins & Comb.	3

**Table 2:** Groups identified at elevated risk by Apriori-SD algorithm

Rule	Prevalence (%)	Risk ratio (95% CI)
Supervision of high-risk pregnancy	25.4	1.29 (1.24–1.35)
Diabetes mellitus	1.4	2.20 (1.96–2.47)
Multiple gestation	2.5	2.11 (1.93–2.32)
Essential hypertension	2.8	1.52 (1.38–1.69)
Known or suspected fetal abnormality affecting management of mother; Other nonspecific abnormal findings	0.3	2.82 (2.28–3.48)
Other conditions or status of the mother complicating pregnancy, childbirth, or the puerperium; Age 35+	2.9	1.39 (1.25–1.55)

## Box 1: Manual grouping of codes identified at 5% FD (maternal codes unless otherwise specified]

* The ICD-10 code (099.411) “Diseases of the circulatory system complicating pregnanecy, fiist trimester” maps in the Centers for Medicare & Medicaid Service; General Equivalence Mapping to 61701 and 61703 which pertain to cerebrovascular disorders in the puerperium.
